# Management of severe crush injury in a front-line tent ICU after 2008 Wenchuan earthquake in China: an experience with 32 cases

**DOI:** 10.1186/cc8160

**Published:** 2009-11-06

**Authors:** Wenfang Li, Jun Qian, Xuefen Liu, Qiang Zhang, Lv Wang, Dechang Chen, Zhaofen Lin

**Affiliations:** 1 Emergency Department, Changzheng Hospital, Second Military Medical University, No. 415 Fengyang Road, Shanghai 200003, China; 2 Intensive Care Unit, The People's Hospital of Jiangyou, No. 346 middle Jinlun Road, Jiangyou City, Sichuan Province, 621700, China

## Abstract

**Introduction:**

The experience on management of crush injury after a devastating earthquake is lacking, and there are even less reports on the front-line critical care of these patients. A front-line intensive care unit (ICU) was set up in a tent after the disastrous Wenchuan earthquake (May, 12, 2008, China), where 32 patients suffering from crush injury were treated from May 12 to May 26. This study summarized our experience on management of 32 crush injury patients in a front-line tent ICU.

**Methods:**

We retrospectively analyzed the clinical data of 32 crush injury patients treated in our frontline tent ICU. Using limited equipment, we observed the arterial blood gas parameters, blood routine, alanine aminotransferase, lactate dehydrogenase, creatine kinase, creatinine, blood urea nitrogen, and urine protein of patients. We also closely watched for changes in crush injury symptoms, urine output, and the dangerous complications of crush injury.

**Results:**

Eighteen of the 32 patients developed traumatic shock, 9 had acute renal failure, 6 had acute heart failure, 2 had stress ulcers and 4 had multiple organ dysfunction syndrome (MODS). The symptoms of 17 patients met the criteria of crush syndrome; hemodialysis and prompt surgical intervention were given to them when necessary. Prompt treatment in our tent ICU improved the symptoms of patients to different degrees. The limb distension and sensory dysfunction were improved and the urine output was increased or even restored to the normal level in some patients. Serological parameters were improved in most patients after admission. Five (15.63%) patients underwent amputation due to severe infection in our group. Six (18.75%) patients died, 4 due to MODS and 2 due to acute renal failure.

**Conclusions:**

Severe crushing injuries and life-threatening complications are major causes of death after major disasters like earthquakes. Prompt treatment and close monitoring of the severe complications are of great importance in saving patients' lives. Establishment of a well-equipped front-line ICU close to the epicentre of the earthquake allows for prompt on the spot rescue of critical patients with crush injury, greatly decreasing the mortality rate and complications and avoiding amputation. There should be sufficient equipment to meet the needs of more patients.

## Introduction

Disasters such as earthquake, debris flow and landslide can cause mass casualties. In addition to direct injuries to vital organs, such as the head and heart and rupture of large vessels, crush injuries caused by prolonged pressing of the body by collapsed buildings are also major causes of death. The acute increase of muscle pressure can lead to compartment syndrome, clinically manifested as progressive swelling of the involved limbs, great pain, diminishing sensory abilities and muscle strength, and even paralysis [1-3]. When exacerbated swelling of body parts, acute renal failure (ARF), shock, or hyperpotassemia is developed, crush syndrome is due to occur. The incidence of crush syndrome is 2% to 15% in all trauma patients, and it can be as high as 30% in earthquake victims. The symptoms of crush syndrome can last for three to five days in mild cases and for one to two weeks in severe cases. About half of the victims develop ARF and the number is almost 100% in those whose symptoms last for 40 hours; among the latter about 50% need hemodialysis. The mortality of patients with crush syndrome can be as high as 40% if the condition lasts for over three weeks [4-8]

The intensive care unit (ICU) is a setting equipped with specially trained medical professionals as well as advanced monitoring system and first aid materials. The aim of an ICU is to monitor and treat patients with critical conditions such as multiple injuries, severe infections, shock of various origins, acute organ failure and disorders of the internal environment of patients. Intensive care reflects the administration proficiency and medical technology advancement of hospitals [9-11]. Close monitoring of pediatric patients [12], aged patients [13] and patients with unstable vital signs can greatly improve their survival rate [14-16]. ICU plays an unreplaceble role in saving the lives of victims after major disasters such as earthquakes, especially those with crush syndrome and complications [17-19]. Demirkiran and colleagues considered that immediate intensive care is vital to the survival of patients with crush injury and compartment syndrome [20]

On 12 May, 2008, a catastrophic earthquake measuring 8.0 on the Richter scale struck the Wenchuan region of Sichuan province, China, causing about 90,000 deaths and even more injuries. The rescue efforts were greatly hampered by the mountainous terrain and damaged roads. Many victims developed critical crush injury and compartment syndrome after their limbs were pressed for a long time during entrapment. Our group, as part of the rescue team of the Second Military Medical University, was sent to Jiangyou city, a severely hit area labouring the most severely struck Beichuan Area. A field hospital was set up in the People's Hospital of Jiangyou, which had been severely damaged during the earthquake. We rescued some undamaged equipment from the severely damaged ICU building and established a front-line tent ICU. From 12 to 26 May, 32 patients with crush injury were treated in our front-line tent ICU. In this paper we reported the treatments and outcomes of the 32 patients and summarize our experience in the front-line tent ICU.

## Materials and methods

### Establishment of the front-line tent ICU

Using the undamaged equipment rescued from the collapsing hospital buildings of the People's Hospital of Jiangyou, we established a front-line tent ICU, as a unit of the field hospital set up by the rescue team of the Second Military Medical University. The ICU had four beds, each equipped with a monitor (DASH3000, GE Company, Connecticut, USA), manual respirator (LVT1000, Newport Corporation, Minnesota, USA) and suction apparatus (YB. DX23D, Shanghai Medicals Corportion, Shanghai, China). Other equipments included a blood filtrum (Prisma Machinegambro, Lund, Swede), a blood gas analyzer (GEMPremier3000, Hartwell, Georgia, USA), a biochemistry inspectoscope (CELLDYN3700, Abbott diagnostics division, Chicago, Illinois, USA), a defibrillator (HEARTSTART XL, PHILIPS, Boblingen, Germany) a trachea cannula and breathing sacculus (GaleMed MR-100, Shanghai Medicals Corportion, Shanghai, China), as well as routine emergency drugs.

### General information of patients

From 12 to 26 May, a total of 32 patients were admitted to our front-line tent ICU, including 21 males and 11 females, with a mean age of 45 ± 19 years (range 13 to 56). Physical examinations upon admission were: the mean body temperature, 37.2 ± 0.6°C (range 36.3 to 37.6); the mean heart rate, 115.3 ± 25.6 beats/min (range 85 to 142); mean respiratory rate, 26.9 ± 5.7 breaths/min (range 21 to 38); mean systolic blood pressure, 121.7 ± 21.3 mmHg (range 78 to 153), and mean diastolic blood pressure, 59.4 ± 16.8 mmHg (range 42 to 96).

### Injuries of patients

Twenty-seven of the 32 patients had multiple injuries and five had lower limb injuries. Nine patients had unilateral lower extremity trauma and 13 had bilateral ones. Three patients had single femoral fractures and seven had bilateral femoral fractures. Thirteen patients were complicated by pelvic fractures, 11 had chest trauma, 8 had cerebral trauma, 6 had splenic rupture, 5 had open tibia fracture, 5 had spinal injuries, and 3 had perinephrium and retroperitoneal hematoma. The mean entrapment period of the patients was 3l ± 12 hours, ranging from 2 to 121 hours. All the patients had swelling and distension of extremities, various degrees of dysesthesia and dyscinesia. Twenty-three patients had soy sauce urine (indicating hemoglobinuria). Seven suffered from anuria and six from pink foam phlegm (a symptom of acute pulmonary edema). The clinical details of the 32 patients are given in Table 1. Informed consents were obtained from each patient or their guardians, and ethical approval was obtained from the Medical Ethics Committee of Changzheng Hospital, the Second Military Medical University.

**Table 1:** Clinical details of the 32 patients in our group

Patient Number	Primary injury	Admitting time	Dark urine	Urine volume (ml)	Protei- nuria	Entrapment time
1	Chest trauma, left humerus fracture and right radius fracture	13 May 2008	√	150	++	3.5

2	Brain trauma and left femur fracture	13 May 2008	√	350	+++	3

3	Fracture of shaft of right femur, pelvic fracture, and splenic rupture	13 May 2008	√	70	++++	5

4	Brain trauma, pelvis fracture, fracture of hypomere of left femur, and right fibula fracture	13 May 2008	√	470	34UY	6

5	Chest trauma, left humeral fracture, right ulna fracture, splenic rupture	13 May 2008		630	++	2.5

6	Left shaft of femur fracture and left tibiofibula fracture	13 May 2008		560	++	4

7	Right femur fracture	13 May 2008	√	480	++	3

8	Brain trauma, pelvic fracture, left femoral neck fracture, and right sprained knee	13 May 2008	√	540	++	4.5

9	Chest trauma, fracture of shaft of left humerus, right ulna and radius fractures	13 May 2008	√	560	++	2.5

10	Brain trauma, hemopneumothorax, left shoulder blade fracture, and sprain of left shoulder joint	13 May 2008		750	++	2

11	Epimere fracture of right shin and sprain of right knee	13 May 2008	√	630	++	3

12	Pelvic fracture, compression fracture of lumber vertebral body, splenic rupture, retroperitoneal hematoma, and left femoral neck fracture	13 May 2008	√	40	++++	5

13	Chest trauma and fractures of shaft of left humerus	13 May 2008	√	450	++	4

14	Pelvic fracture, splenic rupture, perirenal hematoma, fracture of shaft of left femur, and right tibial plateau fracture	13 May 2008	√	90	+++	3.5

15	Brain trauma, splenic rupture, fracture of right shoulder blade, right shoulder joint sprain	13 May 2008		870	++	2

16	Left tibiofibula fractures, compression fractures of lumber vertebral body, and retroperitoneal hematoma	13 May 2008	√	430	++	4

17	Pelvic fracture, fracture of shaft of right femur, and left tibial fracture	13 May 2008		740	++	5.5

18	Chest trauma, and fracture of shaft of left humerus, and right clavicular fracture	13 May 2008	√	510	++	7

19	Pelvic fracture, splenic rupture, right femoral neck fracture, and fracture of left tibial plateau	14 May 2008	√	470	++	6

20	Brain trauma, fracture of lower shaft of femur, and right fibula fracture	14 May 2008	√	540	++	3.5

21	Pelvic fracture, left femoral neck fracture, and right fibula fracture	14 May 2008	√	390	+++	3

22	Chest trauma, fracture of shaft of left humerus, and right ulna fracture	14 May 2008	√	730	++	5

23	Brain trauma, compression fracture of lumber vertebral body, pelvic fracture, and right tibiofibula fracture	14 May 2008	√	70	++++	18

24	Chest trauma, fracture of shaft of left humerus, and right radius fracture	14 May 2008		830	++	9.5

25	Right tibiofibula fracture	14 May 2008	√	460	+++	11

26	Pelvic fracture, compression fracture of choracic12/√lumber1 vertabral body, splenic rupture, retroperitoneal hematoma, fracture of shaft of right femur, and left tibiofibula fracture	15 May 2008	√	60	++++	16

27	Chest trauma, fracture of shaft of right femerus, and left ulna fracture	15 May 2008		850	++	6

28	Chest trauma and fracture of shaft left femerus	15 May 2008		780	++	13

29	Pelvic fracture, splenic rupture, and fracture of right femerus shaft	16 May 2008	√	70	++++	32

30	Brain trauma, pelvic fracture, left inferior femur fracture, and right fibula fracture	16 May 2008	√	490	++	12

31	Fracture of right femur shaft	17 May 2008		670	++	9

32	Chest trauma, fracture of shaft of left femur, and right olecroanon fracture	19 May 2008	√	50	++++	121

### Laboratory tests

Due to the limited equipments, the parameters we could obtain included partial pressure of arterial oxygen, partial pressure of carbon dioxide, PH value, and base excess. Other parameters included blood cell count, serum alanine transaminase (ALT), serum lactate dehydrogenase (LDH), serum creatine kinase (CK), serum creatinine, serum urea nitrogen (BUN), and urine protein. Upon admission the blood test showed the following results:blood hematocrit 39.6 ± 13.4% (range 23 to 52), leukocytes 21,562 ± 8765 cells/μL (range 12,300 to 32,500), platelets 136,775 ± 56,745 cells/μL (range 400,000 to 240,000).

### Diagnosis and treatment of patients with crush syndrome

Crush syndrome is systemic manifestations characterized by swelling and distension of limbs, dyscinesia, myoglobinuria, and hyperpotassemia, usually caused by prolonged pressing of body parts. The mortality rate of patients with crush syndrome could be as high as 50% to 70%. Crush syndrome can be diagnosed when a crush injury patient develops systemic manifestations such as shock, acidosis, and ARF [21-23].

The criteria of crush syndrome in our group were: over one hour pressing of the body parts; involvement of large amount of muscular tissue; development of pallor, clamminess, cold skin, pulselessness, or shock; and the development of manifestations of acute renal failure which are: oliguria less than 400 ml/24 hours, BUN increase more than 40 mg/dl, creatinine increase more than 2 mg/dl, serum potassium increase more than 6 mmol/l, serum phosphorus increase more than 6 mg/dl or serum calcium decrease less than 8 mg/dl. Upon admission, the patients were immediately given interventions such as anti-shock treatment, alkalifying urine, correcting water and electrolyte disturbances, diuresis, dehydration, and anti-infection treatments. Twenty-seven patients received anti-shock treatment, 25 received urine alkalization, 19 received hemodialysis, 15 received fasciotomy, and 5 received amputation due to severe infection. All patients received broad-spectrum antibiotics to control infection and tetanus antitoxin.

Acutely increased interaponeurosis pressure in victims of crush injury can lead to severe muscle necrosis, which requires surgical intervention. Prompt fasciotomy can save lives and prevent the development of dangerous complications after crush syndrome. Indications for fasciotomy included increased turgid of pressed limbs with high tension or/and local ecchymosis, blister in the skin, symptom of 5 "P" (Pain, Pallor, Paralysis, Parathesias, and Pulselessness), persistent urine myoglobin, or interaponeurosis pressure higher than 40 mmHg. Hemodialysis is the first choice for crush syndrome patients complicated with acute renal failure and hyperpotassemia. Indications for hemodialysis included serum creatinine level above 8 mg/dl, BUN above 100 mg/dl, serum potassium above 7 mmol/l, serum bicarbonate below 10 mEq/l, or/and clinical symptoms of ARF, such as edema, hypertension, heart failure, nausea, and vomiting.

### Monitoring of dangerous complications in patients with crush injury

The most important symptom of crush syndrome is acute kidney injury. ARF is defined when a patient with crush injury has one of the following symptoms: oliguria (urine output < 400 ml/24 hours), increases of BUN (> 40 mg/dl), serum creatinine (> 2 mg/dl), uric acid (> 8 mg/dl), potassium (> 6 mmol/l), phosphorus (> 8 mg/dl), or decrease of serum calcium (< 8 mg/dl) [12,13]. We observed the incidence rates of traumatic shock, ARF, acute pulmonary edema (APE), stress ulcer (SU), and multiple-organ dysfunction syndrome (MODS) as well as the vital signs of the patient. Besides, we also closely monitored the changes of urine output, serum BUN, serum creatinine, serum uric acid, urine protein, and serum CK, ALT and LDH. The decrease in amputation rate and morbidity rate were also used to evaluate the outcomes of patients.

### Statistical analysis

All the data were expressed as mean ± standard deviation. Paired t-tests were used when the difference of pre- and post-treatment was in a normal distribution. When the variables did not have a normal distribution and ranked data, the Wilcoxon signed rank sum test was utilized. All data were evaluated using a Microsoft Excel 97 spreadsheet (Microsoft Excel, Seattle, Washington, USA) and SAS9.12 statistical software. Statistical significance was assigned at *P *< 0.05.

## Results

### Improvement of laboratory parameters of patients after intervention

Two weeks after comprehensive treatment, the serum parameters of most patients were greatly improved (Table 2). All the six cases of death had a serum CK level of more than 5000 u/L; two cases of death had a serum potassium level higher than 6.0 mmol/L, which could not be corrected. In the 26 surviving cases the CK value rapidly decreased to below 1000 u/L.

**Table 2:** Improvement of laboratory parameters after treatment in 32 patients with crush injury

	Pre-intervention	Post-intervention	*P*
LDH (u/L)	5725 ± 1859	736 ± 1182	0.000

ALT (u/L)	258 ± 164	69 ± 25	0.000

Potassium (mmol/L)	5.4 ± 2.4	3.8 ± 1.2	0.000

Creatinine (umol/L)	794 ± 85	261 ± 67	0.000

CK (u/L)	4697 ± 359	2281 ± 263	0.000

BUN (mmol/L)	32.6 ± 12.8	12.7 ± 8.7	0.000

Severity of urine protein	++ to ++++	± to +	0.000

ALT = alanine aminotransferase; BUN = blood urea nitrogen;CK = creatine kinase; LDH = lactate dehydrogenase.

### Treatment of complications of patients with crush injury

Of the 32 patients, 18 (56.25%) had traumatic shock, 11 had ARF (34.38%), 6 had APE (18.75%), 2 had SU (6.25%) and 4 had MODS (12.5%); all 4 patients that developed MODS died and the other 26 had improved symptoms. After pertinent treatments, the surviving patients had relieved swelling and distension, and recovered from dysesthesia and anesthesia. Sixteen patients had their dyscinesia symptoms improved and 15 had normal urine output.

A particular case in our group worth further discussion was a 15-year-old girl who had tibial and fibula fracture of her right leg during the earthquake. On admission she had a swelled right leg with fracture blisters on the skin and a decreased pulse of the dorsalis pedis artery. The doctor who first performed the emergent operation for her fractures neglected the risk of crush syndrome. On the next day after fracture fixation, the girl had an acutely reduced urine output (below 100 ml/24 hours) combined with tachypnea, orthopnea, and expectoration of bloody sputum. Auscultation showed moist rales in bilateral lungs. The heart rate (HR) was 140 to 160 beat/min and respiratory rate (RR) was 35 to 46 breaths/min. Pulse oxygen saturation was only 60%. Therefore, she was transferred to our ICU and was diagnosed with crush syndrome accompanied by APE. She was immediately subjected to ventilation by mask oxygen, intravenous injection of cardiotonic, diuretics and hemofiltration. Gradually, the HR and RR of patients decreased and the pulse oxygen saturation was improved.

### Comprehensive treatment of crush syndrome and the outcome of patients

Seventeen (53.13%) of the 32 patients met the diagnosis criterion of crush syndrome. Eighteen (56.25%) patients had traumatic shock, 11 (34.38%) had ARF, 6 had acute heart failure, 2 (6.25%) had stress ulcer, and 4 (12.5%) had MODS. Six (18.75%) patients died in our group, one due to severe capillary leak syndrome, one due to uncontrolled infection after amputation, and four due to MODS. Five (15.63%) patients received amputation due to severe infection of the involved limbs. The 26 surviving patients were alive and well three months later. The major treatment of crush syndrome included anti-shock treatment, surgical intervention and hemodialysis. In total 18 patients received prompt anti-shock treatment and 12 patients were successfully resuscitated. Prompt surgical interventions were given to 15 of the 19 patients who had the indications for fasciotomy; the other four patients did not receive fasciotomy due to severe infection of the wounds. Seventy-two hours later, the limb swelling was aggravated in one of the four patients who did not received fasciotomy initially, and several blisters appeared on the local skin, accompanied by local ecchymosis and decreased artery pulse, indicating increased intramuscular pressure, and fasciotomy was performed finally, but the patient died of MODS. Eleven (34.38%) patients with proper indications received hemodialyses: all of them had different degrees of ARF symptoms, 5 had hyperpotassemia, 7 had anuria, and 4 had combined hyperpotassemia, anuria, and elevated creatinine. The mean urine output of the patient rose from 174.5 ± 82.7 ml to 954.6 ± 132.5 ml after treatment (*P *< 0.05), and the urine output of 15 patients was restored to normal levels.

## Discussion

In this paper we reported the treatment of 32 patients with crush injury in a front-line tent ICU, which was established near the epicenter of the Wenchuan earthquake and was equipped with facilities rescued from the collapsing buildings of a local hospital. Close monitoring and prompt intervention have helped to save the lives of the 26 patients. The tent ICU is of great significance in saving the lives of patients with crush injury following a major disaster. More attention should be given to setting up a well-designed front-line ICU for major disasters.

### Advantages of front-line ICU after an earthquake

A front-line ICU is very important for treatming crush injury patients after disasters such as an earthquake, because it is equipped with advanced facilities and first aid materials needed for critical conditions. A front-line ICU, such as ours, can be located on the site of the disaster, giving treatment in a timely manner [24-26]. It is reported that most victims of disasters and wars died on the spot where they were injured. For example, in a war 40% of the patients died immediately after injury, 25% died 5 minutes after injury, 15% died 5 to 30 minutes after injury, and 20% died 30 minutes after injury; it is indicated that timely treatment of these patients is vital. A front-line ICU can provide this timely treatment, relieve the symptoms of patients, improve their biochemical parameters, and reduce crush syndrome complications, allowing for surgical intervention of the patients. After a major disaster such as Wenchuan earthquake, there will be a large number of patients with crush injury. When compartment syndrome, ARF and/or other severe complications occur, the patients need to be sent to the ICU immediately for closer monitoring. A tent ICU near the epicenter can not only provide timely treatment to the victims, but also avoid the risks of aggravation of patients' condition during the evacuation [27,28].

### Close monitoring, early diagnosis and treatment of crush syndrome

There are a large number of crush injury patients following a major earthquake, and early diagnosis and close monitoring can lower the incidence of crush syndrome. In addition to monitoring the vital signs, more attention should be paid to observing the patient's blood pressure and changes of urine in order to make an early diagnosis of crush syndrome. Observation of the color and volume of urine and the urine protein is also a key step to prevent the transition from crush injury to crush syndrome. Furthermore, monitoring and correcting hypotension can prevent ARF in patients with crush syndrome. In our ICU, only limited biochemical parameters were obtainable; however, close monitoring of the above-mentioned parameters helped us to make early diagnosis and treatment assessments. Due to the limited parameters we could obtain, observation of urine output served as an important parameter for diagnosis of patients and for predication of prognosis. The serum parameter changes caused by muscle necrosis are very important in the diagnosis of crush syndrome. Unfortunately, some important parameters could not be obtained in our ICU due to limited equipment. Sophisticated biochemical instruments are essential for a front-line ICU.

Reportedly, 7 out of 10 patients with crush injury after a catastrophic earthquake developed crush syndrome, and 10% of the total casualty number was due to crush syndrome. Therefore, prevention and management of crush syndrome are critical to lower the mortality rate. The survival rate of our group is 81.25%, greatly higher than that reported previously [29-31].

The major differences of treatments between crush injury and other types of traumas include that patients with crush injury need early and prompt expansion of blood volume to guarantee renal perfusion, correction of acidosis and relief of limb swelling. Most patients with crush injury are in a state of hypotension and need intravenous administration of a large volume of fluids, including artificial plasma, 5% glucose, NaHCO3, aescigenin, and human serum albumin. Colloid should be used to elevate the osmotic pressure and relieve inter-aponeurosis edema; diuretics such as indapamide should be used when circulation is stable. Although mannitol is effective in decreasing inter-aponeurosis pressure, it was not used in our cohort to avoid aggravation of renal function; instead, aescigenin, human serum albumin, and indapamide were used in our patients to relieve swelling of the injured limbs.

### Surgical intervention and post-operation monitoring of patients with crush syndrome

Duman and colleagues believed that prompt fasciotomy in earthquake victims are both life-saving and can prevent some of the severe and dangerous complications of crush syndrome [3]. In fact, not only can close fractures lead to compartment syndrome, but open fractures can also result in it; radical debridement should be performed for open fracture and repeated debridement is needed when necessary. Fasciotomy and expansion of wounds should be performed to remove the necrotic tissues to ensure unobstructed drainage. The aim of fasciotomy is to prevent muscle necrosis, compartment syndrome and the need for amputation. Those who took a negative altitude toward fasciotomy in earthquake victims thought that resection of a large volume of muscle together with the surrounding tissues would inevitably cause loss of fluid and increase the chance of infection. Infection secondary to fasciotomy and primary trauma of earthquake victims have always been grave challenges in the clinic. Ekrem [32] reported that the incidence of severe infection was as high as 37.3% in patients with crush injury. Therefore, in a front-line ICU, the surgical wounds should be closely observed and anti-infection measures should be promptly taken when necessary. In our group, five patients have to receive amputations because of aggravated distension of compressed extremities, deterioration of ecchymosis and blister, local skin becoming purple/black in color, hyperpyrexia and acute increase of leucocytes. Postoperatively, the patients were closely monitored and the incisions were observed. Only one patient of the five died of uncontrollable infection. We believe that surgical intervention of earthquake victims should be considered for earthquake victims when the correct indications are strictly followed.

### Close monitoring of severe complications of crush syndrome

The common complications of crush syndrome include traumatic shock, ARF, acute heart failure, SU, and MODS; early diagnosis and intervention are vital to the survival of patients. In our group, the incidence rates of the aforementioned complications were similar to those reported previously [33-35].

In our front-line tent ICU, energetic anti-shock measures were taken for 18 patients who had traumatic shock to avoid the development of crush syndrome, because many severe fatal complications develop due to long periods of shock. SU is a common manifestation at the final stage of patients in shock and often develops under stress. The incidence rate of SU was reportedly about 4% to 10% in trauma patients [36,37]. Two of our patients suffered from SU. The result indicated that it was necessary to adopt early active mental intervention to relieve the mental stress. The most severe complication of crush injury is MODS. Four of our patients developed MODS and all died. So it is especially important to monitor the functions of major organs to prevent MODS in the front-line ICU. Experience with the 15-year-old girl indicates that APE can also be the first clinical manifestation of crush syndrome; and the necrosis of leg muscle as well as that of the huckle (the part of the leg close to groin) can lead to crush syndrome [38-40].

Eleven of our 32 patients had ARF, a dangerous manifestation of crush syndrome. Hemodialysis is the best choice of treatment for ARF and prevention of crush syndrome. It is reported that hemodialysis can keep the incidence of internal environment disorder and other complications to a minimum. The urine output recovered to normal levels in 11 patients who received hemodialysis. Our ICU only had one hemodialyzer, so patients could not receive continuous hemodialysis and four of our patients developed MODS and died. Advanced portable biochemical analyzer in the front-line ICU allows for close monitor of patients with crush injury, and a blood dialyzer can give prompt, effective treatment to patients who's condition is complicated with ARF [41,42]. When rescuing after a disaster such as the Whenchuan Earthquake, more portable hemodialyzers should be deployed to provide prompt treatment of patients with crush syndrome.

## Conclusions

Severe crush injuries and their life-threatening complications such as crush syndrome are common after a major earthquake like the one that occurred in Wenchuan. The establishment of a front-line ICU close to the epicenter of earthquake allows for a prompt on-spot monitoring and rescue of critical patients suffering from severe traumatic injury, which can decrease the mortality rate and complications in patients with severe crush injury, avoid amputation, and should be encouraged.

## Key messages

• Severe crush injuries and their life-threatening complications such as crush syndrome are common after a major earthquake like the one that occurred in Wenchuan.

• Six (18.75%) patients died in our group, one due to severe capillary leak syndrome, one due to uncontrolled infection after amputation, and four due to MODS. Five (15.63%) patients received amputation due to severe infection of the involved limbs.

• The establishment of a front-line ICU close to the epicenter of the earthquake allows for prompt on-the-spot monitoring and rescue of critical patients suffering from severe traumatic injury, and should be encouraged and studied further.

• In addition to the monitoring of the vital signs, more attention should be paid to observation of the blood pressure and changes of urine to make an early diagnosis of crush syndrome.

• We believe that surgical intervention of earthquake victims should be considered for earthquake victims when the correct indications are strictly followed.

## Abbreviations

ALT: alanine aminotransferase; APE: acute pulmonary edema; ARF: acute renal failure; BUN: blood urea nitrogen; CK: creatine kinase; HR: heart rate; ICU: intensive care unit; LDH: lactate dehydrogenase; MODS: multiple organ dysfunction syndrome; RR: respiratory rate; SU: stress ulcer.

## Competing interests

The authors declare that they have no competing interests.

## Authors' contributions

WL was responsible for data collection, analysis and writing the manuscript. JQ, XL, and QZ participated in data collection and analysis. LW and DC participated in the data collection and revising the manuscript. ZL was responsible for the overall design of the manuscript. All the authors have read and approved the submission.
